# Long-Distance Travellers: Phylogeography of a Generalist Parasite, *Pholeter gastrophilus*, from Cetaceans

**DOI:** 10.1371/journal.pone.0170184

**Published:** 2017-01-13

**Authors:** Natalia Fraija-Fernández, Mercedes Fernández, Kristina Lehnert, Juan Antonio Raga, Ursula Siebert, Francisco Javier Aznar

**Affiliations:** 1 Cavanilles Institute of Biodiversity and Evolutionary Biology, Science Park, University of Valencia, Paterna, Valencia, Spain; 2 Institute for Terrestrial and Aquatic Wildlife Research, University of Veterinary Medicine Hannover, Werftstrasse, Büsum, Germany; National Cheng Kung University, TAIWAN

## Abstract

We studied the phylogeography and historical demography of the most generalist digenean from cetaceans, *Pholeter gastrophilus*, exploring the effects of isolation by distance, ecological barriers and hosts’ dispersal ability on the population structure of this parasite. The ITS2 rDNA, and the mitochondrial COI and ND1 from 68 individual parasites were analysed. Worms were collected from seven oceanic and coastal cetacean species from the south western Atlantic (SWA), central eastern Atlantic, north eastern Atlantic (NEA), and Mediterranean Sea. *Pholeter gastrophilus* was considered a single lineage because reciprocal monophyly was not detected in the ML cladogram of all individuals, and sequence variability was <1% for mtDNA and 0% for ITS2. These results rule out a recent suggestion that *P*. *gastrophilus* would actually be a cryptic-species complex. The genetic cohesion of *P*. *gastrophilus* could rely on the extensive exploitation of wide-ranging and highly mobile cetaceans, with a putative secondary role, if any, of intermediate hosts. Unique haplotypes were detected in SWA and NEA, and an AMOVA revealed significant population structure associated to the genetic variation in these regions. The Equator possibly acts as a significant geographical barrier for cetacean movements, possibly limiting gene flow between northern and southern populations of *P*. *gastrophilus*. A partial Mantel tests revealed that the significant isolation of NEA populations resulted from geographic clustering. Apparently, the limited mobility of cetaceans used by *P*. *gastrophilus* as definitive hosts in this region, coupled with oceanographic barriers and a patchy distribution of potential intermediate hosts could contribute to significant ecological isolation of *P*. *gastrophilus* in NEA. Rather unexpectedly, no genetic differentiation was found in the Mediterranean samples of this parasite. Historical demographic analyses suggested a recent population expansion of *P*. *gastrophilus* in the Atlantic Ocean, perhaps linked to initial association and subsequent spreading in cetaceans.

## Introduction

Digeneans are parasitic organisms that have complex life cycles including free living and parasitic stages, and alternation of asexual and sexual reproduction. Free-living stages, i.e. the miracium and the cercaria, have a limited capacity for dispersal, whereas other stages use invertebrates (usually molluscs) as first intermediate hosts, other invertebrates or vertebrates as second intermediate hosts, and vertebrates as definitive hosts [1]. Consequently, gene flow in digeneans is largely determined by the dispersal ability of the most mobile host, which usually is the definitive host [2]. This is particularly true in the marine environment where few barriers for gene flow presumably exist [3] and, therefore, the dispersal role of hosts is particularly important. Although studies on phylogeography of marine parasites are still scarce [2], evidence suggests contrasting patterns of genetic structure depending on host mobility [4, 5].

Cetaceans harbour a diverse fauna of digeneans and represent an interesting model for phylogeographical analysis because they are long-ranging animals that often undergo seasonal migrations between feeding and breeding grounds [6]. The high dispersal capacity of many oceanic cetaceans results in little genetic differentiation of their populations (e.g., [7, 8, 9]), although geographical barriers may contribute to population structure in some cases [10, 11]. In contrast, cetacean species that are more restricted to neritic waters are known to experience ecological barriers that tend to promote more isolation and local adaptation of populations [12, 13, 14, 15]. How dispersal patterns of cetaceans shape the population structure of their digenean parasites is an open question. As far as we are aware, there is a single study that has investigated the phylogeography and genetic structure of a digenean from cetaceans. Marigo et al. [16] described the genetic structure of *Synthesium pontoporiae*, which is specific to the Franciscana dolphin, *Pontoporia blainvillei*. This largely sedentary cetacean has a relatively restricted geographic range occurring along the temperate coasts of southern Brazil, Uruguay and Argentina. Although there was significant genetic subdivision of Franciscanas at a regional scale [15], no genetic differentiation was found for *S*. *pontoporiae*, which suggests that the dispersal capacity of the (unknown) intermediate hosts would most likely have overcome the low vagility of definitive hosts and contributed to the genetic mixing of *S*. *pontoporiae* populations [16].

The most generalist and geographically widespread digenean exclusive to cetaceans is *Pholeter gastrophilus*, which has been reported in at least 17 species of oceanic and coastal dolphins and porpoises (Table 1). A recent phylogenetic study has confirmed that this species belongs to the family Heterophyidae [17]. *Pholeter gastrophilus* is distributed in the North and South Atlantic Ocean, as well as in the Mediterranean, Black and North Seas (S1 Fig). Remarkably, in the Pacific Ocean just a few reports exist in the Peruvian coast and South Australia and, in the Indian Ocean, sampling is restricted to the Red Sea (S1 Fig). The identity of the intermediate hosts of *P*. *gastrophilus* is unknown but, based on data from other marine heterophyids [1, 18], it can be postulated that eggs are eaten by a gastropod (1st intermediate host), from which cercariae emerge, penetrating and encysting in fish (2nd intermediate host) that serve as prey for cetaceans (definitive host). Evidence indicates that *P*. *gastrophilus* readily infects both sympatric coastal and oceanic dolphins that have largely non-overlapping diets, which would suggest that this parasite is able to extensively exploit the food web to reach its definitive hosts [19].

**Table 1:** Reports of cetacean species infected by the digenean *Pholeter gastrophilus*.

Host species	Habitat	Locality	References
Delphinidae			
*Cephalorhynchus commersonii* (Commerson’s dolphin)	C	AO	[20]
*Delphinus delphis* (Short-beaked common dolphin)	C, O	AO; BS; SA	[21–28] and Pers. Comm. from Ms. Jo Wood (South Australian Museum).
*Globicephala macrorhynchus* (Short-finned pilot whale)	O	AO	[28]
*Globicephala melas* (Long-finned pilot whale)	C, O	AO; MS	[24, 29, 30]
*Grampus griseus* (Risso’s dolphin)	O	AO; MS	[24, 28, 31–33]
*Lagenodelphis hosei* (Fraser’s dolphin)	O	AO	[28]
*Lagenorhynchus acutus* (Atlantic white-sided dolphin)	C, O	AO	[24, 34, 35]
*Lagenorhynchus albirostris* (White-beaked dolphin)	C	AO	[25]
*Lagenorhynchus obscurus* (Dusky dolphin)	C	AO; PO	[36, 37]
*Stenella frontalis* (Atlantic spotted dolphin)	O	AO	[28, 31]
*Stenella coeruleoalba* (Striped dolphin)	O	AO; MS	[19, 22, 28, 38, 39]
*Steno bredanensis* (Rough-toothed dolphin)	O	AO	[28, 40, 41]
*Tursiops aduncus* (Indo-Pacific bottlenose dolphin)	C	RS	[42]
*Tursiops truncatus* (Common bottlenose dolphin)	C, O	AO; PO; MS; BS; SA	[19, 22, 24, 26–28, 43–49] and Pers. Comm. from Ms. Jo Wood (South Australian Museum).
Iniidae			
*Inia geoffrensis* (Amazon river dolphin)	A	A	[50*]
Kogiidae			
*Kogia breviceps* (Pygmy sperm whale)	O	AO	[28]
*Kogia sima* (Dwarf sperm whale)	O	AO	[28]
Phocoenidae			
*Phocoena phocoena* (Harbour porpoise)	C	AO; BS; BaS	[21, 22, 24–26, 45, 51–55]
*Phocoena spinipinnis* (Burmeister’s porpoise)	C	AO; PO	[56, 57]
Physeteridae			
*Physeter macrocephalus* (Sperm whale)	O	AO	[28]
Pontoporiidae			
*Pontoporia blainvillei* (Franciscana)	C	AO	[58]

Available citation reports of cetacean species infected by *Pholeter gastrophilus*. Abbreviations: **A**, Amazon basin; **AO**, Atlantic Ocean; **BaS**, Baltic Sea; **BS**, Black Sea; **C**, Costal; **MS**, Mediterranean Sea; **O**, Oceanic; **PO**, Pacific Ocean; **RS**, Red Sea; **SA**, South Australia. (*) This fresh water record requires further confirmation.

The extensive geographical and ecological distribution of *P*. *gastrophilus* has raised the possibility that it may actually represent a complex of sibling species [38]. Cryptic speciation has been reported in many marine invertebrates with wide geographical distributions [59], including generalist helminths from cetaceans [60] and from other marine vertebrates [61]. For instance, digenean species of *Transversotrema* that infect sedentary fish in the Great Barrier have undergone cryptic speciation associated to limited dispersal of both hosts and parasites [62]. To date, no study has addressed the issue of cryptic speciation in *P*. *gastrophilus*.

Assuming that *P*. *gastrophilus* is a single species, it would be still important to investigate the influence of host mobility in providing genetic structure (or lack of it) to its populations. One could expect that the potentially high dispersal ability of its cetacean hosts could lead to little genetic structure in *P*. *gastrophilus*. This has been observed, for instance, in other geographically widespread marine parasites, such as the acanthocephalan *Profilicollis altmani*, which also infects highly vagile definitive hosts, i.e. marine birds [5]. However, gene flow in *P*. *gastrophilus* could be restricted, at least between some populations, due to limits for host dispersal at vast geographical scales (i.e. thousand km), land barriers between ocean basins, and/or the exploitation of some host species with reduced mobility (i.e. sedentary hosts).

In this study we analysed phylogeographic patterns of *P*. *gastrophilus* using ribosomal and mitochondrial markers. Although collection of specimens was limited by opportunistic sampling, we could obtain samples from a wide geographical range (i.e., South Atlantic, North Atlantic and the Mediterranean Sea), and from cetaceans with contrasting ecology (i.e., oceanic and highly vagile or coastal and sedentary). The research questions we addressed were the following: 1) Does *P*. *gastrophilus* represent one or several species? 2) What is the role (if any) of isolation by distance (Southern vs. Northern Hemisphere), land barriers (Mediterranean vs. Atlantic localities) and host mobility (sedentary vs. highly migratory cetacean species) in providing genetic structure to populations of this species?

## Materials and Methods

### Parasite collection and study design

**Fig 1:**
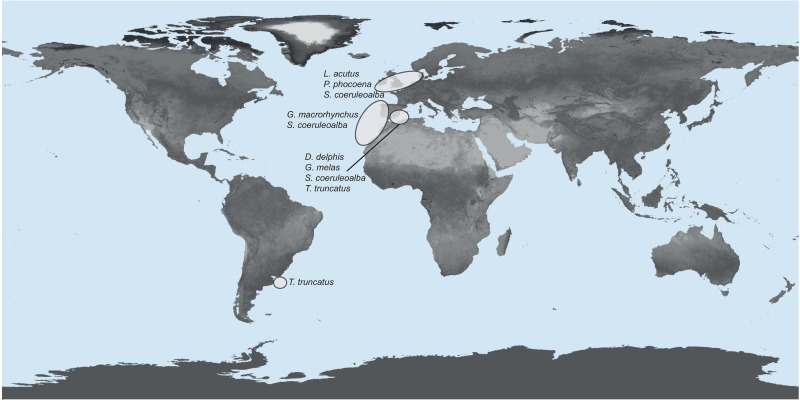
Geographical sampling of the digenean *Pholeter gastrophilus*. Sampling sites of the digenean *Pholeter gastrophilus*, identified by regions and host species. The map has been modified from a NASA image made by Reto Stockli, NASA's Earth Observatory Team, using data provided by the MODIS Land Science Team.

A total of 68 specimens of *P*. *gastrophilus* was collected from the stomach of 55 stranded individuals of seven cetacean species, i.e., striped dolphin, *Stenella coeruleoalba*; bottlenose dolphin, *Tursiops truncatus*; short-beaked common dolphin, *Delphinus delphis*; short-finned pilot whale, *Globicephala macrorhynchus*; long-finned pilot whale, *G*. *melas*; Atlantic white-sided dolphin, *Lagenorhynchus acutus*, and harbour porpoise, *Phocoena phocoena*, in nine localities (Table 2). Permission to collect dead stranded dolphins was given by the wildlife service of regional governments in each locality, specifically the Secretary of Tourism and Wildlife Service of Chubut Province and the Government Agencies of Río Negro and Buenos Aires Provinces (Argentina); the Royal Institute of Natural Sciences of Belgium; the Ministry of Energy, Agriculture, the Environment and Rural Areas of Schleswig-Holstein (Germany); the Department of the Environment, Heritage and Local Government (Ireland), and the Wildlife Services of the Valencian, Andalucian, Galician and Canarian Regional Governments (Spain). No ethics board was involved because animals were collected after their natural death. Worms were collected from the stomach of cetaceans and stored in either 70% or 96% ethanol for further molecular analyses. The nine sampling localities are hierarchically organised into four regions according to their proximity, i.e., 1) Mediterranean Sea (Med), which included samples from the Alboran Sea and the western Mediterranean (Spain); 2) south western Atlantic (SWA), with samples from the coast of Argentina; 3) north eastern Atlantic Ocean (NEA), with samples from the Celtic Sea and the North Sea (Ireland, Belgium and Germany); and 4) central eastern Atlantic (CEA), with samples from the coast of Galicia (north-west Spain), the Strait of Gibraltar, and the Canary Islands (Spain) (Fig 1). Geographical coordinates of each sampling locality are specified in S1 Table. The coast of Galicia was included in the CEA because all worms were collected from short-finned pilot whales, after an exceptional mass stranding event. The distribution of this cetacean species is largely restricted to warm temperate and tropical waters [63].

**Table 2:** Information on the specimens of the digenean *Pholeter gastrophilus* used for this study.

Locality (Country or region)	Host species (Common name)	N (n)	Collection institution
Mediterranean Sea (Alboran Sea and western Mediterranean)	*Globicephala melas* (Long-finned pilot whale)	1 (2)	CEGMA
	*Stenella coeruleoalba* (Striped dolphin)	3 (3)	CEGMA
	*Delphinus delphis* (Short-beaked common dolphin)	1 (2)	ICBIBE
	*Stenella coeruleoalba* (Striped dolphin)	9 (11)	ICBIBE
	*Tursiops truncatus* (Bottlenose dolphin)	5 (7)	ICBIBE
South western Atlantic (Argentina)	*Tursiops truncatus* (Bottlenose dolphin)	1 (3)	CENPAT
Central eastern Atlantic (Canary Islands, Cadiz and Galicia)	*Globicephala macrorhynchus* (Short-finned pilot whale)	3 (5)	CEMMA
	*Stenella coeruleoalba* (Striped dolphin)	10 (11)	CEGMA
	*Stenella coeruleoalba* (Striped dolphin)	1 (1)	ULPGC
North eastern Atlantic (Germany, Belgium and Ireland)	*Phocoena phocoena* (Harbour porpoise)	4 (6)	BMMB
	*Lagenorhynchus acutus* (Atlantic white-sided dolphin)	1 (1)	ITAWR
	*Phocoena phocoena* (Harbour porpoise)	11 (11)	ITAWR
	*Phocoena phocoena* (Harbour porpoise)	2 (4)	SBEES
	*Stenella coeruleoalba* (Striped dolphin)	1 (1)	SBEES

Localities, host species, number of dolphins sampled (N), number of worms collected (n) and institutions responsible for the collection of specimens of the digenean *Pholeter gastrophilus* used for this study. Abbreviations: **BMMB**, Belgian Marine Mammal Biobank, Belgium; **CEGMA**, Centro de Gestión del Medio Marino Andaluz del Estrecho, Spain; **CEMMA**, Coordinadora para o Estudo dos Mamíferos Mariños, Spain; **CENPAT**, Laboratorio de Mamíferos Marinos, Centro Nacional Patagónico, Argentina; **ICBIBE**, Institut Cavanilles de Biodiversitat i Biologia Evolutiva, Spain; **ITAWR**, Institute for Terrestrial and Aquatic Wildlife Research, University of Veterinary Medicine, Germany; **SBEES**, School of Biological, Earth and Environmental Sciences, University College Cork, Ireland; **ULPGC**, University of Las Palmas de Gran Canaria, Spain.

### Molecular analyses: DNA extraction, amplification and sequencing

All collected worms were used for molecular analysis. Prior to DNA extraction, ethanol in the samples was replaced by 500μl of TE buffer (0.001 M TrisHCl, pH 7.5, 0.001 M EDTA, pH 8). Genomic DNA was extracted from individual worms using the QIAGEN DNeasy Blood and Tissue Kit (QIAGEN, Germany), following the manufacturer’s recommendations, except for the incubation period, which was extended overnight. We amplified the internal transcribed spacer 2 (ITS2 rDNA) using primers 3S [64] and ITS2.2 [65]. In addition, the mitochondrial DNA cytochrome *c* oxidase subunit 1 (mtCOI) was amplified using primers JB3 [66] and JB4.5 [67] and the mitochondrial DNA NADH dehydrogenase subunit 1 (mtND1) using primers JB11 [66] and NDJ2a [68]. The thermocycling profile for the ITS2 rDNA amplification was as follows: denaturation at 95°C for 3 min, 40 cycles at 94°C for 50 s, 56°C for 50 s and 72°C for 1 min 20 s, and a final extension at 72°C for 4 min. The profile for the mtCOI amplification was denaturation at 94°C for 5 min, 40 cycles at 92°C for 30 s, 45.6°C for 45 s and 72°C for 90 s, and a final extension at 72°C for 10 min. The profile for mtND1 amplification was denaturation at 95°C for 5 min, 40 cycles at 94°C for 30 s, 50°C for 20 s and 72°C for 45 s, and a final extension at 72°C for 4 min. Amplicons were purified with GFX PCR DNA and Gel Band Purifying Kit (GE Healthcare Life Sciences, UK) and sequenced on an Applied Biosystems ABI 3730 XL automated sequencer by Macrogen Inc. Europe (The Netherlands). Sequencing was done in both directions using PCR primers. Contigs were assembled using BioEdit 7.0.5.3 and sequence identity was checked using the Basic Local Alignment Search Tool (BLAST).

### Phylogenetic analyses

The first dataset used in this study included 68 sequences of the ITS2 rDNA of *P*. *gastrophilus*, and sequences from *Pygidiopsis genata* and *Ascocotyle longa* (Heterophyidae), which were downloaded from GenBank (accession numbers AY245710 and AY245703, respectively) and used as outgroups. Outgroups were selected according to previous phylogenetic studies for the family Heterophyidae [17]. Sequences were aligned using the online version of MAFFT, available at http://mafft.cbrc.jp/alignment/server/. Complete alignment was analysed and uncorrected pairwise *p* distance matrices were obtained using MEGA 6 [69].

An additional dataset included 68 concatenated sequences of each corresponding mtND1 and mtCOI sequence from each worm, and sequences of *Metagonimus yokogawai* (Heterophyidae) and *Opisthorchis felineus* (Opisthorchiidae), which were downloaded from GenBank (accession numbers NC011127 and NC023249, respectively) and used as outgroups. Prior to concatenation, mitochondrial sequences were aligned independently for each gene using the online version of MAFFT, and were analysed using JModelTest 4.1.2 [70]. The Hasegawa, Kishino and Yano model with gamma distribution (HKY+G) was selected as the best model that fit both, mtCOI and mtND1 alignments independently. Sequences were concatenated and the complete dataset was run on MEGA 6 [69] for a ML analysis under the selected model of evolution. Bootstrap values were obtained after 1000 replicates and the heuristic tree searching strategy was Subtree-Pruning-Regrafting (SPR).

### Genetic diversity analyses

For population analyses, the 68 sequences of the concatenated regions of the mtND1 and mtCOI of *P*. *gastrophilus* were arranged according to the four geographic regions defined previously, i.e., Med, SWA, CEA and NEA (Fig 1). Genetic diversity for the complete dataset (n = 68) and for each population were estimated as the number of unique haplotypes, the number of segregating or polymorphic sites (S), haplotype diversity (Hd) (i.e., the probability that two randomly sampled haplotypes are different), and nucleotide diversity (π) (i.e., the average number of nucleotide differences per site), without considering missing data, as implemented in DNAsp [71]. A haplotype network using Minimum Spanning Tree was constructed to illustrate the connections between haplotypes using Population Analysis with Reticulate Trees (PopART) available at http://popart.otago.ac.nz [72].

Pairwise genetic distances for the aligned mitochondrial dataset were calculated with PAUP* [73] under the HKY model of evolution. To examine whether genetic differentiation in samples from *P*. *gastrophilus* might be influenced by a pattern of isolation by distance, Mantel tests were explored as implemented in the library VEGAN from the public domain statistical software R [74, 75]. Genetic distances were calculated as Φ_ST_ between pairs of localities in the Isolation by Distance Web Service, available at http://ibdws.sdsu.edu/~ibdws [76]. Geographic distances, expressed as nautical miles (nm), were estimated to the nearest port using the online platform http://ports.com. An effect of isolation by distance would be indicated, prima facie, by a positive and significant correlation between geographic and genetic distances. However, the Mantel test cannot differentiate patterns resulting from geographical clustering, from those generated by isolation by distance where there is equal migration among populations [77, 78]. Therefore, a partial Mantel test was used to explore whether the correlation between genetic and geographic distances of samples was affected by a spatially structured effect. For the partial Mantel tests a third matrix was included in the correlation with the information on the geographical structuring of localities, i.e., whether pairs of localities were from the same region or not. We are aware of the recent debate on the probability of type I error in partial Mantel tests (see [79]), thus results were interpreted with caution, and the null hypothesis of isolation by distance was rejected only if *p* was < 0.001 [80, 78]. In the partial Mantel test we permuted localities within regions [77], thus samples from SWA were excluded from the analysis because all samples were collected from a single locality. A graphical representation of the pattern of isolation by distance was made using the Isolation by Distance Web Service [76].

### Population structure analyses

The partitioning of genetic variation within sequences of *P*. *gastrophilus* was investigated with an Analysis of Molecular Variance (AMOVA) [81]. AMOVA estimates the genetic variance at different levels of a hierarchical division of the population and test the null hypothesis of no genetic structure between regions using a permutational approach [81]. The power for detection of genetic structure between regions decreases with fewer samples per locality in the population [82]. Following Fitzpatrick’s [82] approach, we estimated the number of possible permutations and the minimum *p* value according to the region structure designed for *P*. *gastrophilus*, i.e., one locality for SWA, two localities for Med, three localities for CEA, and three localities for NEA (see Fig 1). The genetic structure in *P*. *gastrophilus* was partitioned into variation from all samples considering the nine localities (“within the population” Φ_ST_ estimate), the variation among four regions, i.e., SWA, NEA, Med and CEA (“among regions” Φ_CT_ estimate) and the variation among localities within regions (“among localities, within regions” Φ_SC_ estimate). To assess “among regions” variation, three alternative scenarios were considered according to geographic proximity and the need of generating an acceptable number of possible permutations. First, we examined the relative importance of isolation by distance with two scenarios, (1) samples from the Celtic Sea were grouped with CEA (i.e., the AMOVA included one locality for SWA, two localities for Med, four localities for CEA and two localities for NEA); (2) samples from SWA were grouped with CEA (i.e., four localities for SWA+CEA, two localities for Med and three localities for NEA). Second, we assessed the relative importance of land barriers by grouping samples from the Strait of Gibraltar with Med (i.e., one locality for SWA, three localities for Med, two localities for CEA and three localities for NEA). *P* values associated with the fixation indices were evaluated through 2520 and 1260 random permutations for a four-grouped and a three-grouped structure, respectively, as implemented in Arlequin v. 3.5. [83].

### Demographic history analyses

Tajima’s D and Fu’s tests were used to assess whether sequences of *P*. *gastrophilus* departed from the assumption of neutrality [84, 85]. Positive or negative values of Tajima’s D and Fu’s would imply the operation of non-neutral processes (e.g., balancing selection or selective sweep, respectively), whereas significant values would be consistent with population expansion [84]. Tajima’s D and Fu’s tests and their significance values were calculated with Arlequin v. 3.5, based on 1000 simulated re-sampling replicates [83]. The significant threshold for Tajima’s D was set at 0.05, and for Fu’s at 0.02 [83, 85]. The R2 neutrality test was also used because it seems to be more powerful for detecting population growth with small sample sizes [86, 87]. R2 values and its significance, under 10,000 coalescent simulations, were calculated with the Population and Evolutionary Genetics Analysis System (PEGAS), implemented in the software R [75, 88].

Growth or decline episodes in a population leave signals in the distribution of pairwise nucleotide differences between pairs of individuals, which can be analysed through a mismatch distribution [89]. Unimodal and smooth wave-like distributions suggest recent population expansion, whereas irregular multimodal distributions would occur in stationary or shrinking populations [90]. Mismatch distribution was computed in Arlequin v.5.3, and the smoothness of the observed distribution was quantified with the Harpending’s Raggedness Index under 10,000 bootstrap replicates.

## Results

### Phylogenetic analyses

We obtained 68 sequences from the ITS2 rDNA from specimens of *P*. *gastrophilus*, which were between 407 base pairs (bp) and 501 bp long. Pairwise distance comparison for the aligned portion of the ITS2 rDNA indicated no genetic variation among specimens of *P*. *gastrophilus* across the sampled regions.

**Fig 2:**
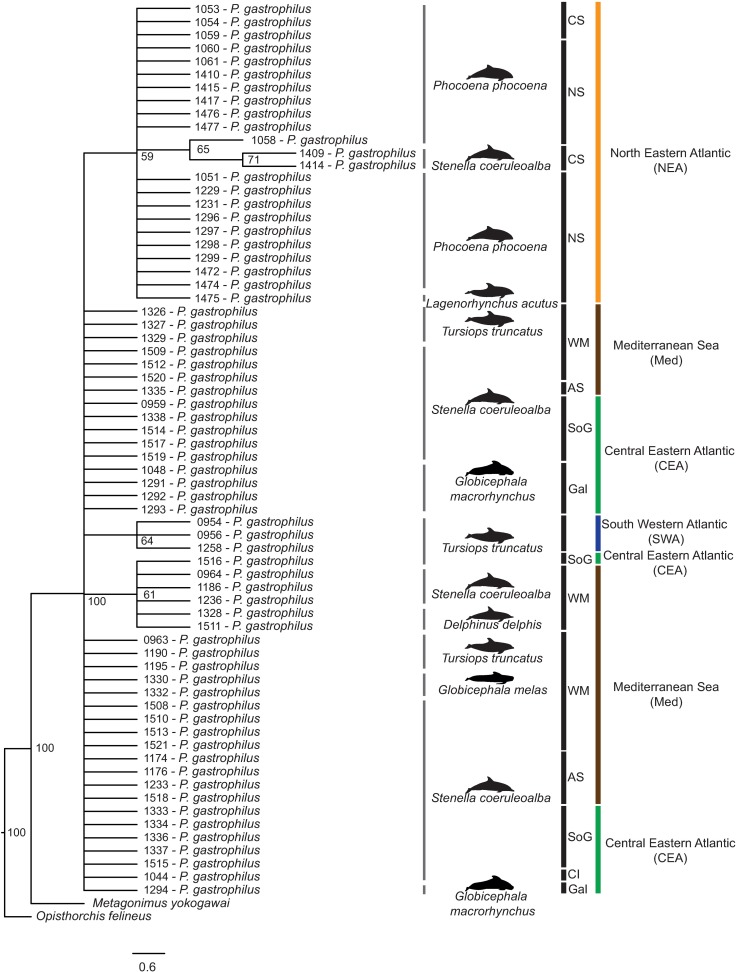
Maximum Likelihood analysis. Analysis for the phylogenetic relationships inferred from the mtND1 and mtCOI sequences of 68 individuals of the digenean *Pholeter gastrophilus*, collected from seven host species and four geographical regions. Support values for each node were calculated as ML bootstrap after 1000 replicates. Sequences are labelled according to their sampling localities as follows: central eastern Atlantic, CEA (Canary Islands, CI; Galicia North-West Spain, Gal; Strait of Gibraltar, SoG); Mediterranean Sea, Med (Alboran Sea, AS; western Mediterranean, WM); north eastern Atlantic, NEA (Celtic Sea, CS; North Sea, NS); south western Atlantic, SWA.

The 68 mtND1 sequences of *P*. *gastrophilus* were between 407 bp and 504 bp long, whereas the 68 mtCOI sequences were between 341 bp and 441 bp long. The alignment of the concatenated sequences of the mtND1 and the mtCOI was 701 bp long excluding sites with missing data, determined by the length of the shortest available sequence. The ML hypothesis suggests that all worms collected from the four regions corresponded to a single and well-supported lineage (ML bootstrap = 100%; Fig 2). The intraspecific topology of the ML hypothesis did not support reciprocally monophyletic groups of samples. No internal node received bootstrap support ≥ 71%, although some clades received support ≥ 59%, including samples for NEA (59%) and SWA (64%). An additional clade was also found, grouping five and one sequences from Med and CEA, respectively (ML bootstrap = 61%) (Fig 2).

### Genetic diversity analyses

**Fig 3:**
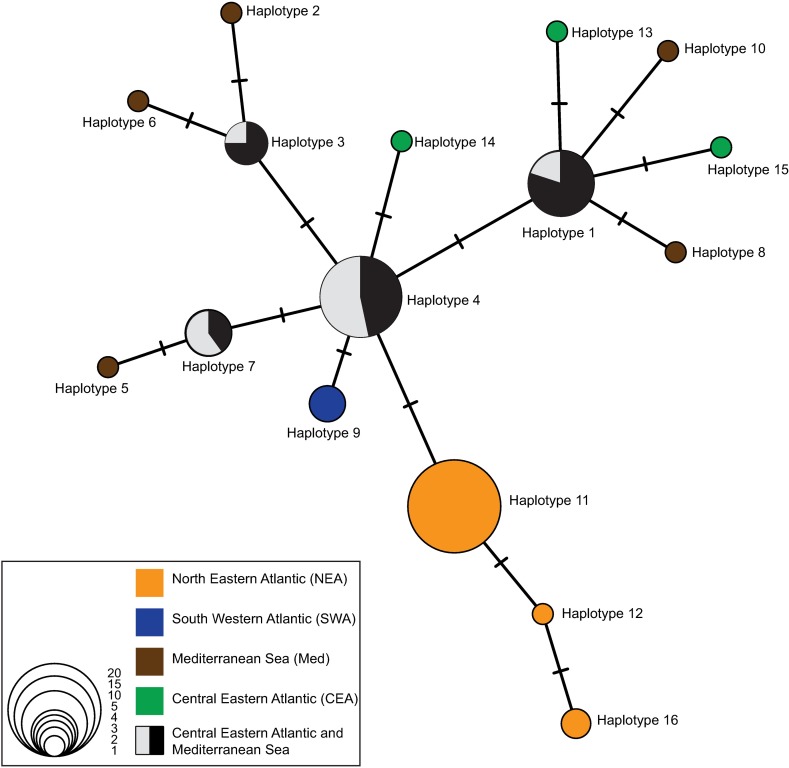
Haplotype network. Minimum Spanning network for the 16 haplotypes identified for the digenean *Pholeter gastrophilus* in this study. Each haplotype is labelled according to corresponding numbers in Table 3. Lines located transversally to connecting branches represent the number of base pair changes between haplotypes. Size and colour of circles match the information at the bottom-left side of the figure.

A total of 15 polymorphic sites and 16 different haplotypes were found in the 701 bp-alignment of the concatenated mtND1 and mtCOI sequences (Table 3). The haplotype network showed a relatively simple, star-like topology (Fig 3). Haplotype 4, occurring in Med and CEA, was located centrally. Three haplotypes, namely 1, 3 and 7, stemmed from haplotype 4 and were also shared between Med and CEA. Five haplotypes occurred exclusively in the Med population (haplotypes 2, 5, 6, 8 and 10, see Fig 3), where the highest haplotype and nucleotide diversity was observed (Hd = 0.82; π = 0.0021, Table 4), followed by the CEA, with three exclusive haplotypes, i.e., 13, 14 and 15 (Fig 3), and Hd = 0.76; π = 0.0016. Three unique haplotypes stemming from the central haplotype were found among individuals of NEA (haplotypes 11, 12 and 16, Fig 3; Hd = 0.24; π = 0.0006), whereas a single haplotype was found in individuals from SWA (haplotype 9, Fig 3; Hd = 0; π = 0). Single mutational steps established the connection between all haplotypes. The mtND1 showed the highest number of polymorphic sites, representing 14 out of the 16 defined haplotypes, whereas only two haplotypes were found in the mtCOI (data not shown).

**Table 3:** GenBank accession numbers of haplotypes identified for the digenean *Pholeter gastrophilus*.

Region	Haplotype no.	Frequency	GenBank accession no.
Mediterranean Sea (Med)	2	1	KX059398
	5	1	KX059401
	6	1	KX059402
	8	1	KX059404
	10	1	KX059406
Central eastern Atlantic (CEA)	13	1	KX059409
	14	1	KX059410
	15	1	KX059411
South western Atlantic (SWA)	9	3	KX059405
North eastern Atlantic (NEA)	11	20	KX059407
	12	1	KX059408
	16	2	KX059412
Med + CEA	1	10 (Med: 8, CEA: 2)	KX059397
	3	4 (Med: 3, CEA: 1)	KX059399
	4	15 (Med: 7, CEA: 8)	KX059400
	7	5 (Med: 2, CEA: 3)	KX059403

Frequency and GenBank accession numbers of the 16 haplotypes identified for the mtND1 and mtCOI sequences of the digenean *Pholeter gastrophilus* in different geographic regions.

**Table 4:** Genetic diversity indices for the digenean *Pholeter gastrophilus*.

Region	N	S	H	Hd	Π
Mediterranean Sea (Med)	25	8	9	0.82	0.0021
Central eastern Atlantic (CEA)	17	6	7	0.76	0.0016
North eastern Atlantic (NEA)	23	2	3	0.24	0.0006
South western Atlantic (SWA)	3	0	1	0.00	0.0000
TOTAL	68	15	16	0.84	0.0023

Number of polymorphic sites (S), number of haplotypes (H), haplotype diversity (Hd), and nucleotide diversity (π) for the digenean *Pholeter gastrophilus* in different geographical regions. “N” is the sample size. Note that four haplotypes were shared between Med and CEA, hence the total H = 16.

**Fig 4:**
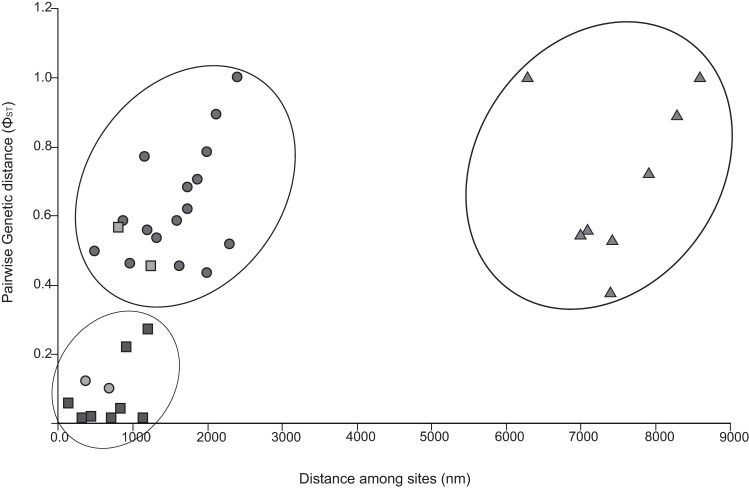
Isolation by distance analysis. Graphical representation of the isolation by distance analysis among populations of the digenean *Pholeter gastrophilus*. The relationship between pairwise genetic distance (Φ_ST_) and geographical distance (nm) between sampling sites are shown. Triangles represent comparisons of samples from the south western Atlantic (SWA); squares represent comparison of samples with the Mediterranean Sea (Med) and the central eastern Atlantic (CEA); and circles represent comparison of samples with the north eastern Atlantic (NEA).

Genetic pairwise distances for the concatenated mitochondrial sequences (mtND1 and mtCOI) were 0.2% (CEA vs. Med and SWA vs. CEA) and 0.3% (SWA vs. Med, and NEA vs. all localities). The plot for the correlation between genetic and geographic distances is shown in Fig 4. There was a significant pattern of isolation by distance, suggesting an increase of genetic distances together with geographic distances (Mantel test, r = 0.515; *p* < 0.001). However, when the spatial structure of localities was considered a non-significant partial Mantel test was obtained (Partial Mantel test, r = 0.438; *p* = 0.009), suggesting that the geographical clustering of localities, and not the isolation by distance, would account for the pattern observed in samples from the CEA, Med and NEA.

### Population structure analyses

The AMOVA showed significant genetic differentiation in *P*. *gastrophilus* at different scales. The highest amount of variation was found within the population, which explained 59.71% of the overall variation (Φ_ST_ = 0.40, *p* < 0.001, Table 5). A significant “among regions” variation (Φ_CT_ = 0.37, *p* = 0.005) was also found, which accounted for 37.1% of the overall variation. However, only 3.2% of the overall variation was explained by “among localities, within regions” variation (Φ_SC_ = 0.05, *p* = 0.129) (Table 5). In an alternative arrangement, we grouped samples of the Celtic Sea with those from CEA, and “among localities, within regions” variation increased to 10.3%, and the “among regions” variation decreased to 29.1%. A similar pattern was observed when sequences of SWA were grouped with CEA. In this case, the “among localities, within regions” variation increased to 11.1% and the “among regions” variation decreased to 28.7%. When sequences from the Strait of Gibraltar (SoG) were grouped with Med, the “among localities, within regions” variation remained low (2.3%) and the “among regions” variation still accounts for a sustainable percentage of the overall variation (41.3%) (Table 5). Overall, these results suggest that the genetic variation in the population of *P*. *gastrophilus* is mainly accounted for by samples from NEA and SWA.

**Table 5:** Analysis of Molecular Variance (AMOVA) of sequences of mtND1 and mtCOI from 68 individuals of the digenean *Pholeter gastrophilus*.

1) Geographical regions				
**Med** (AS + WM); **SWA** (Arg); **CEA** (Gal + SoG + CI); **NEA** (CS + NS)				
Source of variation	d.f.	Percentage of variation (%)	Fixation indices	*p* value
Among regions	3	37.14	Φ_CT_ 0.37	0.005
Within regions	5	3.15	Φ_SC_ 0.05	0.129
Within population	59	59.71	Φ_ST_ 0.40	<0.001
2) Geographical regions				
**Med** (AS + WM); **SWA** (Arg); **CEA** (Gal + CI + SoG + CS); **NEA** (NS)				
Source of variation	d.f.	Percentage of variation (%)	Fixation indices	*p* value
Among regions	3	29.11	Φ_CT_ 0.29	0.023
Within regions	5	10.25	Φ_SC_ 0.14	0.011
Within population	59	60.65	Φ_ST_ 0.39	<0.001
3) Geographical regions				
**Med** (AS + WM); **SWA + CEA** (Arg + Gal + CI + SoG + CS); **NEA** (CS + NS)				
Source of variation	d.f.	Percentage of variation (%)	Fixation indices	*p* value
Among regions	2	28.73	Φ_CT_ 0.28	0.025
Within regions	6	11.12	Φ_SC_ 0.15	0.001
Within population	9	60.15	Φ_ST_ 0.39	<0.001
4) Geographical regions				
**Med** (AS + WM + SoG); **SWA** (Arg); **CEA** (Gal + CI); **NEA** (CS + NS)				
Source of variation	d.f.	Percentage of variation (%)	Fixation indices	*p* value
Among regions	3	41.28	Φ_CT_ 0.41	<0.001
Within regions	5	2.34	Φ_SC_ 0.04	0.161
Within population	59	56.38	Φ_ST_ 0.44	<0.001

Three scenarios are shown according to different arrangements of geographical regions. Abbreviations: **Arg**, Argentina coast; **AS**, Alboran Sea; **CEA**, central eastern Atlantic; **CI**, Canary Islands; **CS**, Celtic Sea; **Gal**, Galicia; **Med**, Mediterranean Sea; **NEA**, north eastern Atlantic; **NS**, North Sea; **SoG**, Strait of Gibraltar; **SWA**, south western Atlantic; **WM**, western Mediterranean.

### Demographic history analyses

**Fig 5:**
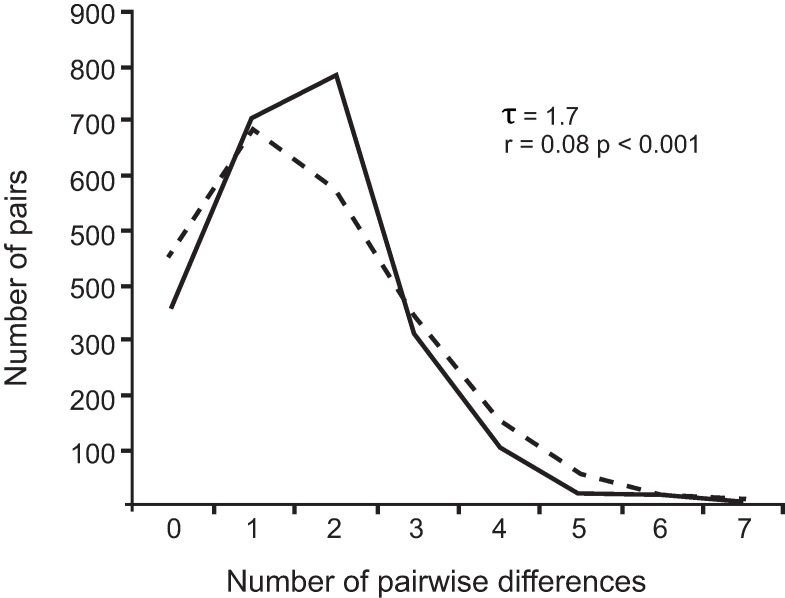
Mismatch distribution. Mismatch distribution curve for the digenean *Pholeter gastrophilus* showing the expected (dashed line) and the observed values (continuous line) under the expanding population model. The time of expansion in units of mutational time (τ) and the Harpending’s Raggedness Index (r) are shown.

Values from both Tajima’s D (-1.45, *p* = 0.042) and Fu’s (-9.22, *p* < 0.001) tests were negative and statistically significant. The R2 was low and significant (R2 = 0.049, *p* = 0.031). Overall, these results suggest that sequences from *P*. *gastrophilus* have a bias towards few segregating sites and few haplotypes compared to what would be expected from the neutral theory, supporting a population expansion of *P*. *gastrophilus*. Although a significant Harpending’s Raggedness Index (*p* < 0.001) for the mismatch distribution would reject the null hypothesis of population expansion, our results showed a clearly unimodal distribution (Fig 5) which, along with the star-like haplotype network, the low value of Harpending’s Raggedness Index (r = 0.08) and the results from the neutrality tests, agree with a population expansion model. In addition, the pattern of distribution and the small value of τ (τ = 1.7) indicate that the population expansion was recent and started from a small population (θ = 0).

## Discussion

Our results support the hypothesis that all populations of *P*. *gastrophilus* surveyed in this study represent a single species. No variation was found in the ITS2 rDNA sequences from all samples, and a maximum genetic difference of just 0.3% was detected in the mtND1 and mtCOI between worms from the two most distant regions (SWA vs. NEA). Previous studies have suggested that the maximum intraspecific variation in digeneans to recognise separate species would be ~1% for ITS2 rDNA [91] and ~5% for mtDNA [92], well above the observed values for *P*. *gastrophilus*. The use of this “genetic yardstick” has been criticised (see, e.g., [93]), and reciprocal monophyly has been considered as a suitable alternative method for species delimitation [93–95]. In our study, the absence of reciprocal monophyly confirms that *P*. *gastrophilus* is a single lineage, with no deep genetic differentiation associated to specific geographical and/or ecological factors. In any event, sample sizes of *P*. *gastrophilus* are modest in some localities and, therefore, more sequences from the South Atlantic, and new samples from the Pacific Ocean, would provide additional support for the hypothesis of a single species.

As noted above, the free-living stages of digeneans have low dispersal ability, thus genetic exchange between their populations must rely on the vagility of their hosts [2, 4]. Since the identity of the intermediate hosts of *P*. *gastrophilus* is unknown [96], we use data from other heterophyids to draw inferences on the role of these hosts in the dispersal of *P*. *gastrophilus*. All known first intermediate hosts of heterophyids belong to three superfamilies of bottom-dwelling snails, i.e., Cerithioidea, Rissooidea and, more rarely, Littorinoidea [97, 98]. Given that digeneans exhibit strong phylogenetic conservatism in the use of first intermediate hosts [18], it is likely that the life cycle of *P*. *gastrophilus* also includes some of these benthic gastropods, although their specific identity may likely vary depending on the geographical region. For instance, the liver fluke, *Fasciola hepatica*, always use lymnaeid snails as first intermediate hosts, but the particular species differs, e.g., between Europe and Australia [18]. Interestingly, *P*. *gastrophilus* infects both coastal and oceanic cetaceans (Table 1), suggesting that its first intermediate host could have a broad bathymetric range; in fact, several cerithioid and rissooid snails do [99]. This scenario is not unusual in marine digeneans; for instance, *Brachyphallus crenatus* infects a benthic gastropod that reaches depths up to 500 m [99, 100], and also includes a wide array of neritic and oceanic teleosts as definitive hosts [97]. On the other hand, the second intermediate hosts of heterophyids are generally fish, which become infected through skin penetration of free-swimming cercariae [18]. In agreement with this observation, all cetaceans in which *P*. *gastrophilus* has been reported are at least partially piscivorous [101], but it is unclear how many fish species could function as second intermediate hosts. In any event, we would hardly expect that fish, or other cetacean prey, could act as paratenic hosts for *P*. *gastrophilus* since paratenicity is exceptional in digeneans, and has never been described in heterophyids [1].

According to the above discussion, the genetic cohesion found among populations of *P*. *gastrophilus* would mostly rely on the extensive exploitation of wide-ranging and highly mobile cetaceans. The ability of pelagic cetaceans to move over long distances has been documented for several species that serve as hosts for *P*. *gastrophilus*, including, e.g., the short-beaked common dolphin [8, 102], the oceanic populations of the bottlenose dolphin [103, 104], the dusky dolphin [105], the short-finned pilot whale [106, 107], and the sperm whale [108] (Table 1). However, we detected significant population structure in the Atlantic population of *P*. *gastrophilus*, which was mainly accounted for samples from the two most distant regions, i.e., SWA and NEA. Sequences of worms from SWA shared a unique haplotype and formed a single, though low supported, clade in the ML cladogram. The small sample size precluded the possibility of exploring whether this difference was linked to the existence of geographical or ecological barriers or simply resulted from isolation by distance. Interestingly, though, in the southern Atlantic *P*. *gastrophilus* occurs in Argentinean waters but is virtually absent in Brazil (S1 Fig). This patchy distribution may perhaps indicate that the life cycle cannot be completed in low latitudes, and so the genetic connectivity between North and South Atlantic populations of *P*. *gastrophilus* would depend on trans-equatorial movements of cetaceans [109, 110]. Although there are records of tracked individuals that have undergone long-distance migrations (e.g. Risso’s dolphin [111], bottlenose dolphin [112], or striped dolphin [39]), the Equator possibly acts as a significant geographical barrier for cetacean movements, possibly limiting gene flow between northern and southern populations of *P*. *gastrophilus*.

A single clade and three unique haplotypes were detected for specimens from NEA, and the AMOVA indicated that the genetic structure in *P*. *gastrophilus* might partly be attributed to differences between the NEA and all other regions. Also, results from the partial Mantel test indicated that these differences could primarily result from the existence of geographical and/or ecological barriers isolating NEA samples. A certain degree of isolation of *P*. *gastrophilus* in this area could partly be accounted for by the ecological distribution of its cetacean hosts. Out of the 23 samples collected from the NEA, 21 were from a largely sedentary, coastal cetacean, i.e., the harbour porpoise [12], and other records of *P*. *gastrophilus* in the NEA are mostly from coastal cetaceans as well (S2 Table). Further ecological factors could reinforce isolation of the NEA populations of *P*. *gastrophilus*. In particular, the southern Bay of Biscay functions as a transition zone between the boreal and the subtropical regions, generating an oceanographic boundary for movements of marine organisms [113], including some cetaceans [12, 114, 115]. It is also worth noting that *P*. *gastrophilus* has never been reported in typical boreal cetaceans (including harbour porpoises) from the Atlantic Iberian coast (S1 Fig). This gap in the distribution of *P*. *gastrophilus* could also hamper genetic mixing through stepping-stone processes [5] between northern and southern populations in the NE Atlantic.

Rather unexpectedly, we found little genetic differentiation between samples of *P*. *gastrophilus* from Med and CE Atlantic. The Strait of Gibraltar generates, not only a physical barrier separating the Mediterranean Sea and the Atlantic Ocean, but also a thermal front (the Almeria-Orán Front) situated ca. 350 km eastward from the Strait [116]). Both factors, particularly the Front, are known to limit gene flow in some fish and mollusc species [117, 118]. In addition, there is evidence of genetic distinctiveness of western Mediterranean populations of at least two cetacean species that serve as hosts for *P*. *gastrophilus*, namely, the striped dolphin and the bottlenose dolphin [11, 13, 119]. At least in the latter, it is believed that the Almeria-Orán Front contributes to isolation because the dolphin populations are locally adapted to feed on specific prey from each side of the Front [13]. We hypothesize that the genetic connectivity of *P*. *gastrophilus* across the Strait of Gibraltar would depend on regular in-and-out movements of some cetaceans, or perhaps fish intermediate hosts, that are not affected by oceanographic barriers. For instance, populations of the common dolphin, a typical host for *P*. *gastrophilus*, exhibit ample gene flow between the Mediterranean Sea and Atlantic Ocean, apparently because they chase pelagic prey that are highly vagile [8].

The neutrality tests rendered negative and significant values, suggesting that the population of *P*. *gastrophilus* has suffered either an expansion or an evolutionary event that removed variation in the population (e.g., selective sweeps or bottleneck events) [84]. Note that Fu’s and R2 tests are more powerful than Tajima’s [87], particularly when sample size is small [86]. This pattern is compatible with the hypothesis that *P*. *gastrophilus* suffered a population bottleneck in the Atlantic Ocean. Alternatively, the observed demographic structure could reflect the origin and subsequent spreading of *P*. *gastrophilus* in cetaceans, at least in the Atlantic Ocean. It has been hypothesized that the ancestor of *P*. *gastrophilus* colonised cetaceans from marine birds via trophic guilds [17]. Since colonization via host switching should start from a small population, the population of *P*. *gastrophilus* would have had undergone an expansion by exploiting different cetacean species. This hypothesis, however, would imply that the origin of the association between *P*. *gastrophilus* and cetaceans occurred in the Atlantic, its occurrence in the Pacific basin being more recent (Table 1, S1 Fig). This hypothesis should be tested in the future by including samples from the entire geographic range of *P*. *gastrophilus*, and based on molecular data from more than one locus using a coalescent framework (see [120] for a review).

In summary, results from this study indicate that there is ample genetic connectivity among populations of *P*. *gastrophilus*, but also significant genetic structure at regional scale. Both features would depend on (1) the dispersal patterns of the most mobile (definitive) host, which are in part influenced by oceanographic barriers, and (2) perhaps the patchy distribution of potential intermediate hosts at a global scale. There is positive evidence of this connectivity for some parasites infecting marine birds [5], but there are many unexplored host-parasite systems that deserve a closer look, especially those involving large pelagic vertebrates such as elasmobranchs or marine turtles. A broader implication of our study is that the mobility of definitive hosts might be a major factor shaping the genetic structure of other trophically-transmitted, generalist marine parasites that are geographically widespread.

## Supporting Information

S1 Fig Worldwide distribution of *Pholeter gastrophilus*. Geographically distributed surveyed localities for *P*. *gastrophilus*. The number outside parentheses is the amount of individual cetacean species surveyed for *P*. *gastrophilus*; the number in parentheses is the number of infected dolphins; and the number in square brackets indicates the bibliographic source. Complete references are included.
(PDF)

S1 Table Geographic coordinates of collection sites. Geographic coordinates of each collection site of *Pholeter gastrophilus*.
(DOCX)

S2 Table Reported prevalences of *Pholeter gastrophilus* within the distribution of the species. Prevalences of *P*. *gastrophilus* within the distribution of the species, including the number of infected hosts and the sampling size for each region. Note that the habitat of the host species is detailed. Complete references are at the end of the table.
(XLSX)
