# Supplementary material for: Long-Distance Travellers: Phylogeography of a Generalist Parasite, Pholeter gastrophilus, from Cetaceans
Source: PLoS One. 2017 Jan 13;12(1):e0170184. doi: 10.1371/journal.pone.0170184 (PMC5234839; doi:10.1371/journal.pone.0170184)
Supplement: S1 Fig — Geographically distributed surveyed localities for P. gastrophilus. The number outside parentheses is the amount of individual cetacean species surveyed for P. gastrophilus; the number in parentheses is the number of infected dolphins; and the number in square brackets indicates the bibliographic source. Complete references are included. (PDF) [file pone.0170184.s001.pdf]

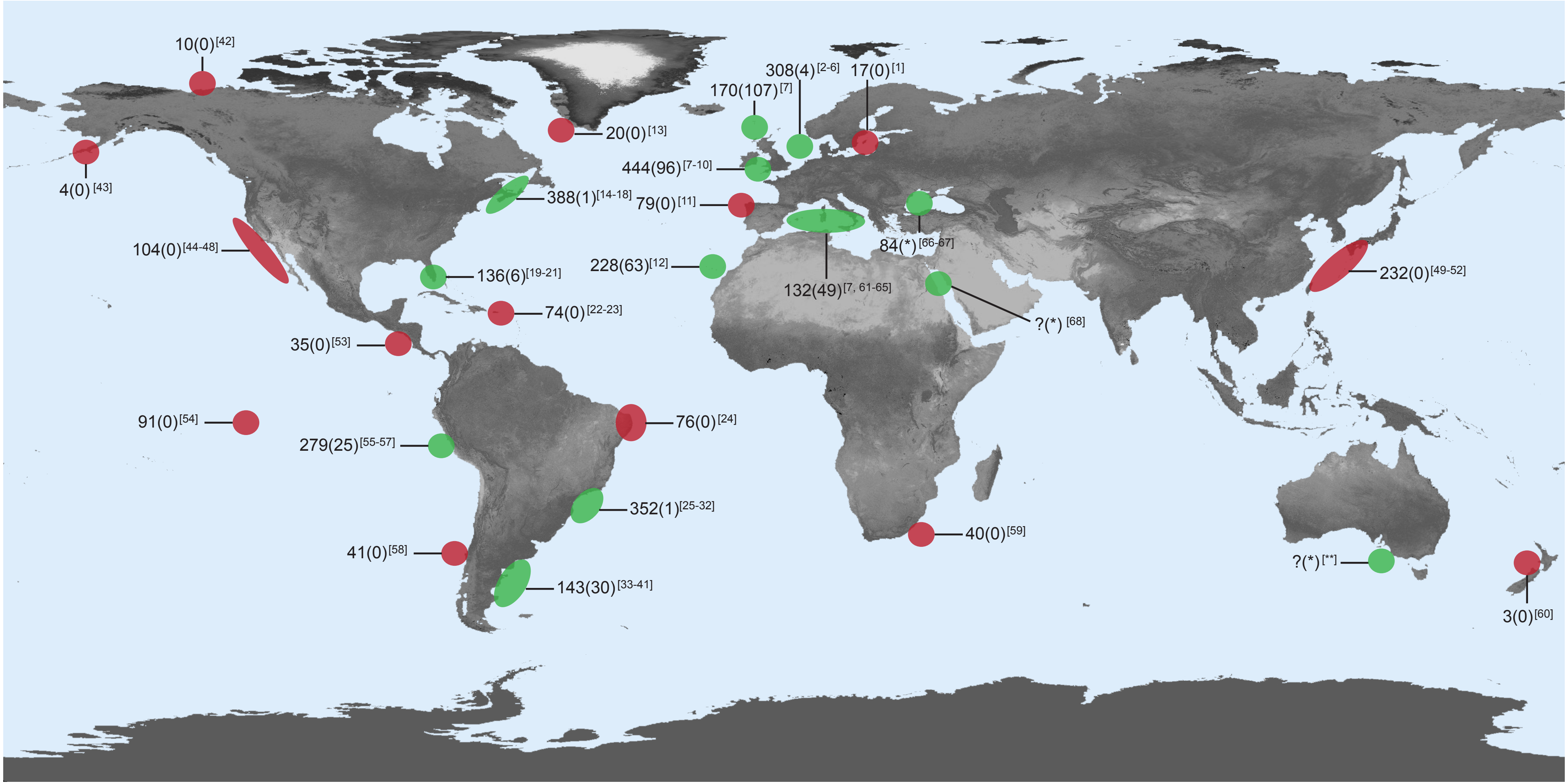

S1 Fig. Worldwide distribution of *Pholeter gastrophilus*. Geographically distributed surveyed localities for *P. gastrophilus*. The number outside parentheses is the amount of individual cetacean species surveyed for *P. gastrophilus*; the number in parentheses is the number of infected dolphins; and the number in square brackets indicates the bibliographic source. Complete references are included.

Boreal Atlantic
Baltic Sea = [1]
North Sea = [2-6]
Feroe Islands = [7]
United Kingdom and Ireland = [7-10]
Galicia (Spain) = [11]
Canary Islands (Spain) = [12]
West Greenland = [13]
North West Atlantic = [14-18]

Tropical Atlantic
Florida = [19-21]
Puerto Rico and Virgin Islands = [22-23]
Northeastern Brazil = [24]
Southern Brazil = [25-32]

Austral Atlantic
Patagonia (Argentina) = [33-41]

Boreal Pacific
Canada = [42]
Alaska (USA) = [43]
Oregon and California (USA) = [44-48]
Hong Kong (China) and Japan = [49-52]

Tropical Pacific
Costa Rica = [53]
Eastern Tropical Pacific = [54]
Peru = [55-57]

Austral Pacific
Chile = [58]
South Africa = [59]
New Zealand = [60]

Mediterranean and Black Sea
Mediterranean Sea = [7, 61-65]
Black Sea = [66-67]

Red Sea
Egypt = [68]

? Information on the number of sampled hosts is missing.

(*) Information on the number of infected hosts is missing; only presence of *P. gastrophilus* is reported.

[**] Personal communication from Ms. Jo Wood and Leslie Chisholm (Parasitology Collection Manager, South Australian Museum).

REFERENCES

[1] Rokicki J, Berland B, Wróblewski J. Helminths of the harbour porpoise, *Phocoena phocoena* (L.), in the southern Baltic. Acta Parasitol. 1997; 42: 36-39.

[2] Brosens L, Jauniaux T, Siebert U, Benke H, Coignoul F. Observations on the helminths of harbour porpoises (*Phocoena phocoena*) and common guillemots (*Uria algae*) from the Belgian and German coasts. Vet. Rec. 1996; 139: 254-257.

[3] Clausen B, Andersen S. Health status of harbour porpoise (*Phocoena phocoena*) from Danish waters. Dan. Rev. Game Biol. 1988; 13: 1-20.

[4] Herreras MV, Kaarstad SE, Balbuena JA, Kinze CC, Raga JA. Helminth parasites of the digestive tract of the harbour porpoise *Phocoena phocoena* in Danish waters: a comparative geographical analysis. Dis. Aquat. Org. 1997; 28: 163-167.

[5] Lehnert K, Raga JA, Siebert U. Macroparasites in stranded and bycaught harbour porpoises from German and Norwegian waters. Dis. Aquat. Org. 2005; 64: 265-269.

[6] Raga JA, Kinze CC, Balbuena JA, Ortiz T, Fernández M. New data on helminth parasites of the harbour porpoise *Phocoena phocoena* in Danish waters. Proceedings of the 3rd Annual Conference of the European Cetacean Society, La Rochelle, France, 24-26 Februrary; 1989. pp. 88-90.

[7] Aznar FJ, Fognani P, Balbuena JA, Pietrobelli M, Raga JA. Distribution of *Pholeter gastrophilus* (Digenea) within the stomach of four odontocete species: the role of the diet and digestive physiology of hosts. Parasitology. 2006; 133: 369–380.

[8] Baker JR, Martin AR. Causes of mortality and parasites and incidental lesions in harbour porpoises (*Phocoena phocoena*) from British waters. Vet. Rec. 1992; 130: 554-558.

[9] Gibson DI, Harris EA, Bray RA, Jepson PD, Kuiken T, Baker JR, Simpson VR. A survey of the helminth parasites of cetaceans stranded on the coast of England and Wales during the period 1990-1994. J. Zool. 1998; 244: 563-574.

[10] Rogan E, Baker JR, Jepson PD, Berrow S, Kiely O. A mass stranding of white-sided dolphins (*Lagenorhynchus acutus*) in Ireland: biological and pathological studies. J. Zool. 1997; 242: 217-227.

[11] Abollo E, López A, Gestal C, Benavente P, Pascual S. Macroparasites in cetaceans stranded on the northwestern Spanish Atlantic coast. Dis. Aquat. Org. 1998; 32: 227-231.

[12] Díaz-Delgado J. Patología y causas de la muerte de los cetáceos varados en las Islas Canarias (2006-2012). Ph.D. Thesis, Universidad de Las Palmas de Gran Canaria. 2015. Available from: http://hdl.handle.net/10553/17258.

[13] Lehnert K, Seibel H, Hasselmeier I, Wohlsein P, Iversen M, Nielsen NH, et. al. Increase in parasite burden and associated pathology in harbour porpoises (*Phocoena phocoena*) in West Greenland. Polar Biol. 2014; 37: 321-331.

[14] Beverley-Burton B. Helminths of the alimentary track from a stranded herd of the Atlantic white-sided dolphin, *Lagenorhynchus acutus*. J. Fish Res. Board Can. 1978; 35: 1356-1359.

[15] Brattey J, Stenson GB. Helminth parasites of the alimentary tract of the harbour porpoise, *Phocoena phocoena* (L.) from Newfoundland and Labrador. Proc. Helminthol. Soc. Wash. 1995; 62, 209-216.

[16] Cowan DF. Helminth parasites of the pilot whale *Globicephala melaena* (Traill 1809). J. Parasitol. 1967; 53: 166-167.

[17] Lair S, Measures LN, Martineau D. Pathologic findings and trends in mortality in the Beluga (*Delphinapterus leucas*) population of the St Lawrence Estuary, Quebec, Canada, from 1983 to 2012. Vet. Pathol. 2015; 53: 22-36.

[18] Measures LN, Béland P, Martineau D, De Guise S. Helminths of an endangered population of belugas, *Delphinapterus leucas*, in the St. Lawrence estuary, Canada. Can. J. Zool. 1995; 73: 1402-1409.

[19] Forrester DJ, Robertson WD. Helminths of rough-toothed dolphins, *Steno bredanensis* Lesson 1828, from Florida waters. J. Parasitol. 1975; 61, 922.

[20] Woodard JC, Zam SG, Caldwell DK, Caldwell MC. Some parasitic diseases of dolphins. Vet. Pathol. 1969; 6: 257−272.

[21] Zam SG, Caldwell DK, Caldwell MC. Some endoparasites from small odontocete cetaceans collected in Florida and Georgia. Cetology. 1971; 2: 1-11.

[22] Colón-Llavina MM, Mignucci-Giannoni A, Mattiucci S, Paoletti M, Nascetti G, Williams EH. Additional records of metazoan parasites from Caribbean marine mammals, including genetically identified anisakid nematodes. Parasitol. Res. 2009; 105: 1239-1252.

[23] Mignucci-Giannoni A, Hoberg EP, Siegel-Causey D, Williams EH. Metazoan parasites and other symbionts of cetaceans in the Caribbean. J. Parasitol. 1998; 84: 939-946.

[24] Carvalho VL, Leal Bevilaqua CM, Mayo Iñiguez A, Mathews-Cascon H, Bezerra Ribeiro F, Bezerra Pessoa LM, et al. Metazoan parasites of cetaceans off the northeastern coast of Brazil. Vet. Parasitol. 2010; 173: 116-122.

[25] Andrade A, Pinedo C, Barreto AS. Gastrointestinal parasites and prey items from a mass stranding of false killer whales, *Pseudorca crassidens*, in Rio Grande do Sul, Southern Brazil. Rev. Bras. Biol. 2001; 61: 55-61.

[26] Andrade A, Pinedo C, Pereira J. The gastrointestinal helminths of the Franciscana, *Pontoporia blainvillei*, in Southern Brazil. Rep. Int. Whal. Commn. 1997; 47: 669-673.

[27] Domiciano IG, Domit C, Broadhurst MK, Koch MS, Bracarense APFRL. Assessing disease and mortality among small cetaceans stranded at a world heritage site in Southern Brazil. PLoS One. 2016; 11(2): e0149295.

[28] Marigo J, Rosas FCW, Andrade ALV, Oliveira MR, Dias RA, Catao-Dias JL. Parasites of franciscana (*Pontoporia blainvillei*) from São Paulo and Paraná States, Brazil. LAJAM. 2002; 1: 115-122.

[29] Marigo J, Ruoppolo V, Rosas FCW, Valente ALS, Oliveira MR, Dias RA, et al. Helminths of *Sotalia guianensis* (Cetacea: Delphinidae) from the South and Southeastern Coasts of Brazil. J. Wildl. Dis. 2010; 46: 599-602.

[30] Melo OP, Ramos RMA, Di Beneditto APM. Helminths of the marine tucuxi, *Sotalia fluviatilis* (Gervais, 1853) (Cetacea: Delphinidae), in northern Rio de Janeiro State, Brazil. Braz. Arch. Biol. Technol. 2006; 49: 145–148.

[31] Santos CP, Rohde K, Ramos R, Di Beneditto AP. Helminths of cetaceans on the Southeastern coast of Brazil. Proc. Helminthol. Soc. Wash. 1996; 63: 149-152.

[32] Valente AL, Ebert M. First record of *Pholeter gastrophilus* (Digenea: Heterophyidae) in Brazilian waters. Proceedings of the X Congreso de la Sociedad Latinoamericana de Especialistas en Mamíferos Acuáticos (SOLAMAC), Cartagena, Colombia, 1–5 December; 2014.

[33] Aznar FJ, Balbuena JA, Raga JA. Helminth communitites of *Pontoporia blainvillei* (Cetacea: Pontoporiidae) in Argentinian waters. Can. J. Zool. 1994; 72: 702-706.

[34] Berón-Vera B, Pedraza SN, Raga JA, Gil de Pertierra A, Crespo EA, Alonso MK, et al. Gastrointestinal helminths of Commerson's dolphins *Cephalorhynchus commersonii* from central Patagonia and Tierra del Fuego. Dis. Aquat. Org. 2001; 47: 201-208.

[35] Berón-Vera B, Crespo EA, Raga JA, Fernández M. Parasite communities of common dolphins (*Delphinus delphis*) from Patagonia: the relation with host distribution and diet and comparison with sympatric hosts. J. Parasitol. 2007; 93: 1056-1060.

[36] Berón-Vera B, Crespo EA, Raga JA. Parasites in stranded cetaceans of Patagonia. J. Parasitol. 2008; 94: 946-948.

[37] Capozzo HL, Negri MF, Mahler B, Lía VV, Martínez P, Gianggiobe A, et al. Biological data on two Hector's beaked whales, *Mesoplodon hectori*, stranded in Buenos Aires Province, Argentina. LAJAM. 2005; 4: 113-128.

[38] Corcuera J, Monzón F, Aguilar A, Borrell A, Raga JA. Life history data, organochlorine pollutants and parasites from eight Burmeister's porpoises, *Phocoena spinipinnis*, caught in Northern Argentine waters. Rep. Int. Whal. Commn. 1995; 16: 365-372.

[39] Dans SL, Reyes LM, Pedraza SN, Raga JA, Crespo EA. Gastrointestinal helminths of the Dusky dolphin, *Lagenorhynchus obscurus* (Gray, 1828), off Patagonia, in the Southwestern Atlantic. Mar. Mamm. Sci. 1999; 15: 649-660.

[40] Fernández M, Berón-Vera B, García N, Raga JA, Crespo EA. Food and parasites from two Hourglass dolphins, *Lagenorhynchus cruciger* (Quoy and Gaimard, 1824), from Patagonian waters. Mar. Mamm. Sci. 2003; 19: 832-836.

[41] Romero MA, Fernández M, Dans SL, García NA, González R, Crespo EA. Gastrointestinal parasites of bottlenose dolphins *Tursiops truncatus* from the extreme southwestern Atlantic, with notes on diet composition. Dis. Aquat. Org. 2014; 108: 61-70.

[42] Wazura KW, Strong JT, Glenn CL, Bush AO. Helminths of the beluga whale (*Delphinapterus leucas*) from the Mackenzie River Delta, Northwest Territories. J. Wildl. Dis. 1986; 22: 440-442.

[43] Shults LM, Fay FH, Hall JD. Helminths from Stejneger's beaked whale *Mesoplodon stejnegeri* and Risso's dolphin *Grampus griseus* in Alaska. Proc. Helminthol. Soc. Wash. 1982; 49: 146-147.

[44] Aguilar-Aguilar R, Moreno-Navarrete RG, Salgado-Maldonado G, Villa-Ramírez B. Gastrointestinal helminths of spinner dolphins *Stenella longirostris* (Gray, 1828) (Cetacea: Delphinidae) stranded in La Paz Bay, Baja California Sur, Mexico. Comp. Parasitol. 2001; 68: 272-274.

[45] Dailey MD, Stroud R. Parasites and associated pathology observed in cetaceans stranded along the Oregon coast. J. Wildl. Dis. 1978; 14: 503-511.

[46] Dailey MD. Distribution of helminths in the Dall porpoise (*Phocoenoides dalli* True). J. Parasitol. 1971; 57: 1348.

[47] Dailey MD. Baseline data on parasites from marine mammals in Southern California. In Marine mammals and seabird survey of the Southern California Bight area. Vol. III. Principal investigator's reports. Book 1. Pinnipedia, Cetacea and Parasitology. Santa Cruz: University of California; 1978.

[48] Johnston DG, Ridgway SH. Parasitism in some marine mammals. J. Am. Vet. Med. Assoc. 1969; 155: 1064-1072.

[49] Kuramochi T, Kikuchi T, Okamura H, Tatsukawa T, Doi H, Nakamura K, et al. Parasitic helminth and epizoit fauna of finless porpoise in the Inland Sea of Japan and the Western North Pacific with a preliminary note on faunal difference by host's local population. Mem. Natl. Sci. Mus. (Tokyo). 2000; 33: 83-95.

[50] Miyazaki N, Fujise Y, Iwata K. Biological analysis of a mass stranding of melon-headed whales (*Peponocephala electra*) at Aoshima, Japan. Mem. Natl. Sci. Mus. (Tokyo). 1998; 24: 31-60.

[51] Parsons ECM, Overstreet RM, Jefferson TA. Parasites from Indo-Pacific hump-backed dolphins (*Sousa chinensis*) and finless porpoises (*Neophocaena phocaenoides*) stranded in Hong Kong. Vet. Rec. 2001; 23: 776-780.

[52] Tajima Y, Maeda K, Yamada TK. Pathological findings and probable causes of the death of Stejneger's beaked whales (*Mesoplodon stejnegeri*) stranded in Japan from 1999 and 2011. J. Vet. Med. 2015; 77: 45-51.

[53] Oliveira JB, Morales JA, González-Barrientos RC, Hernández-Gamboa J, Hernández-Mora G. Parasites of cetaceans stranded on the Pacific coast of Costa Rica. Vet. Parasitol. 2011; 182: 319-328.

[54] Dailey MD, Perrin WF. Helminth parasites of porpoises of the genus *Stenella* in the Eastern Tropical Pacific, with descriptions of two new species: *Mastigonema stenellae* gen. et. sp. n. (Nematoda: Spiruroidea) and *Zalophotrema pacificum* sp. n. (Trematoda: Digenea). Fish. Bull. 1973; 71: 455-471.

[55] Reyes JC, Van Waerebeek K. Aspects of the biology of Burmeister's porpoise from Peru. Rep. Int. Whal. Commn. 1993; 16: 349-364.

[56] Tantalean M, Cabrera R. Algunos helmintos de la marsopa espinosa, *Phocoena spinipinnis* de la Reserva Nacional de Paracas, Perú. Parasitol. al día 1999; 23: 56-57.

[57] Van Waerebeek K, Reyes JC, Alfaro J. Helminth parasites and phoronts of dusky dolphins *Lagenorhynchus obscurus* (Gray 1828) from Peru. Aquat. Mamm. 1993; 19: 159-169.

[58] Torres P, Oporto JA, Brieva LM, Escare L. Gastrointestinal helminths of the cetaceans *Phocoena spinipinnis* (Burmeister, 1865) and *Cephalorhynchus eutropia* (Gray, 1846) from the Southern coast of Chile. J. Wildl. Dis. 1992; 28: 313-315.

[59] Lane EP, de Wet M, Thompson P, Siebert U, Wohlsein P, Plön S. A systematic health assessment of Indian Ocean bottlenose (*Tursiops aduncus*) and Indo-Pacific humpback (*Sousa plumbea*). Plos ONE. 2014; 9: e107038.

[60] McKenzie J, Blair D. Parasites from Hector's dolphin (*Cephalorhynchus hectori*). New Zeal. J. Zool. 1983; 10: 126-127.

[61] Cornaglia E, Rebora L, Gili C, Di Guardo G. Histopathological and immunohistochemical studies on cetaceans found stranded on the coast of Italy between 1990 and 1997. J. Vet. Med. A 2000; 47: 129–142.

[62] Fernández M, Agustí C, Aznar FJ, Raga JA. Gastrointestinal helminths of Risso's dolphin *Grampus griseus* from the Western Mediterranean. Dis. Aquat. Org. 2003a; 55: 73-76.

[63] Fernández M, Aznar FJ, Montero FE, Georgiev BB, Raga JA. Gastrointestinal helminths of Cuvier's beaked whales, *Ziphius cavirostris*, from the western Mediterranean. J. Parasitol. 2004; 90: 418-420.

[64] Manfredi MT, Dini W, Ganduglia S, Podesta M, Repetto G. Parasitological findings in striped dolphins *Stenella coeruleoalba*. Proceedings of the 6th Annual Conference of the European Cetacean Society, San Remo, Italy 20–22 February; 1992. pp. 218-219.

[65] Raga JA, Carbonell E. New data about parasites on *Stenella coeruleoalba* (Meyen, 1833) (Cetacea: Delphinidae) in the western Mediterranean Sea. Invest. Cetacea. 1985; 17: 207–213.

[66] Aytemiz I, Dede A, Danyer E, Tonay A. Morphological identification of parasites found in the stomach contents of bycaught striped dolphins (*Stenella coeruleoalba*) from Turkish Eastern Mediterranean Sea coast. J. Black Sea/Mediterranean Environment. 2012; 18: 238-245.

[67] Delyamure SL. Towards the study of the helminth fauna of the dolphin, *Tursiops tursio*. J Fish Res Board Can 1959; 134: 1-5.

[68] Kleinertz S, Hermosilla C, Ziltener A, Kreicker S, Hirzmann J, Abdel-Ghaffar F, et al. Gastrointestinal parasites of free-living Indo-Pacific bottlenose dolphins (*Tursiops aduncus*) in the Northern Red Sea, Egypt. Parasitol. Res. 2014; 113: 1405-1415.
