# Supplementary material for: Long-Distance Travellers: Phylogeography of a Generalist Parasite, Pholeter gastrophilus, from Cetaceans
Source: PLoS One. 2017 Jan 13;12(1):e0170184. doi: 10.1371/journal.pone.0170184 (PMC5234839; doi:10.1371/journal.pone.0170184)
Supplement: S1 Table — Geographic coordinates of each collection site of Pholeter gastrophilus. (DOCX) [file pone.0170184.s002.docx]

**S1 Table. Geographic coordinates of collection sites.** Geographic coordinates of each collection site of *Pholeter gastrophilus*.

| **Region** | **Locality** | **Collection site** | **Coordinates** |
| --- | --- | --- | --- |
| Mediterranean Sea | Alboran Sea | Malaga (Spain) | 36º43’N, 4º23’W |
|  |  | Marbella (Spain) | 36º30’N, 4º53’W |
|  |  | Motril (Spain) | 36º42’N, 3º29’W |
|  | Western Mediterranean | Benicassim (Spain) | 40º03’N, 0º04’E |
|  |  | Benidorm (Spain) | 38º32’N, 0º07’W |
|  |  | Denia (Spain) | 38º50’N, 0º05’E |
|  |  | Elche (Spain) | 38º15’N, 0º31’W |
|  |  | Guardamar del Segura (Spain) | 38º05’N, 0º38’W |
|  |  | Nules (Spain) | 39º49’N, 0º06’W |
|  |  | Sagunto (Spain) | 39º39’N, 0º12’W |
|  |  | Torrevieja (Spain) | 37º58’N, 0º39’W |
| South Western Atlantic | Argentina | San Antonio Oeste (Argentina) | 40º45’S, 64º57’W |
| North East Atlantic | Celtic Sea | Cork (Ireland) | 51º45’N, 8º18’W |
|  | North Sea | Büsum (Germany) | 54º07’N, 8º50’E |
|  |  | Dagebüll (Germany) | 54º40’N, 8º16’E |
|  |  | Ostende (Belgium) | 51º13’N, 2º54’E |
|  |  | Sankt Peter-Ording (Germany) | 54º17’N, 8º35’E |
|  |  | Sylt (Germany) | 54º54’N, 8º16’E |
| Central Eastern Atlantic | Canary islands | Las Palmas (Spain) | 28º07’N, 15º27’W |
|  | Galicia | O Vicedo (Spain) | 43º44’N, 7º40’W |
|  | Strait of Gibraltar | Tarifa (Spain) | 36º00’N, 5º36’W |
